# Transmission chains of the first local outbreak cause by Delta VariantB.1.617.2 COVID-19 in Guangzhou, Southern China

**DOI:** 10.1186/s12879-023-08819-3

**Published:** 2024-01-02

**Authors:** Chun Chen, Ke Li, Yong Huang, Chaojun Xie, Zongqiu Chen, Wenhui Liu, Hang Dong, Shujun Fan, Lirui Fan, Zhoubin Zhang, Lei Luo

**Affiliations:** Guangzhou Center for Disease Control and Prevention, 1# Qide Road, Guangzhou, Guangdong 510440 China

**Keywords:** COVID-19, Delta variant, Outbreak, Transmission chain, Superspreading event

## Abstract

**Background:**

The first local outbreak of Delta Variant B.1.617.2 COVID-19 of China occurred in Guangzhou city, south China, in May 2021. This study analyzed the transmission chains and local cluster characteristics of this outbreak, intended to provide information support for the development and adjustment of local prevention and control strategies.

**Methods:**

The transmission chains and local cluster characteristics of 161 local cases in the outbreak were described and analyzed. Incubation period, serial interval and generation time were calculated using the exact time of exposure and symptom onset date of the cases. The daily number of reported cases and the estimated generation time were used to estimate the effective reproduction number (Rt).

**Results:**

We identified 7 superspreading events who had more than 5 next generation cases and their infected cases infected 70.81%(114/161) of all the cases transmission. Dining and family exposure were the main transmission routes in the outbreak, with 29.19% exposed through dining and 32.30% exposed through family places. Through further analysis of the outbreak, the estimated mean incubation period was 4.22 (95%CI: 3.66–4.94) days, the estimated mean generation time was 2.60 (95%CI: 1.96–3.11) days, and the estimated Rt was 3.29 (95%CI: 2.25–5.07).

**Conclusions:**

Classification and dynamically adjusted prevention and control measures had been carried out according to analysis of transmission chains and epidemical risk levels, including promoting nucleic acid screening at different regions and different risk levels, dividing closed-off area, controlled area according to the risk of infection, raising the requirements of leaving Guangzhou. By the above control measures, Guangzhou effectively control the outbreak within 28 days without implementing a large-scale lockdown policy.

## Background

Delta variant known as a new variant of SARS-CoV-2 was first detected in India in December 2020, and became the most commonly reported variant in the country starting in mid**-**April 2021 [1]. As of May 192,021, the variant had been detected in 43 countries across six continents in GISAID [2]. 21 May to 19 June 2021there were a total of 161 local confirmed COVID-19 cases infected by COVID-19 Delta Variant B.1.617.2 in Guangzhou city, Guangdong province, southern China. Since the first case was detected on 21 May 2021, and the transmission relationship of all the 161 cases were started with this case. This first case and the 161 caseswere the first local confirmed case and the first local outbreak of COVID-19 Delta Variant B.1.617.2 occurred in China.For this study, we analyzed the transmission chains and local cluster characteristics of this COVID-19 Delta Variant B.1.617.2 outbreaks.

## Methods

### Study design

The transmission chains and the epidemical characteristics of the outbreak were described and analyzed using a cross-sectional study design.

### Data source

All the 161 cases were laboratory-confirmed via nasopharyngeal swab testing by reverse transcription (RT-PCR) and diagnosedas SARS-CoV-2 positive according to “Guidelines for the Diagnosis and Treatment of Novel Coronavirus (SARS-CoV-2) Infection by the National Health Commission (Trial Version 7)”.In accordance with requirements in the Guidance for COVID-19: Prevention, Control, Diagnosis and Management, information about all COVID-19 outbreaks and cases should be reported to the China Information System for Disease Control and Prevention.

In our study, clusters were defined as two or more confirmed infections with close contact, superspreading events(SSEs) were defined as the 99^th^ percentile of the offspring distribution for the number of secondary cases caused by a primary case [3].

### Statistical analysis

Using the exact time of exposure and symptom onset date, we obtained a range of values for the incubation period (the time lag between exposure and the onset of symptoms of a case), serial interval (the time lag between the onset of symptoms of primary case and secondary case), and generation time(the time lag between infection of primary case and secondary case) of each case. Then we used Weibull, Gamma and Lognormal distributions methods to estimate the probability distribution of the corresponding incubation period, serial interval and generation time obtained by taking the primary-secondary casepairs (incubation period: 41 pairs, serial interval: 58 pairs, generation time: 30 pairs) with a clear transmission chain. The AIC criterion was used to determine the optimal distribution. The mean and 95% confidence interval of parameters was calculated with 1000 bootstrap simulations from the optimal distribution [4].

### The effective reproduction number (Rt)

The daily number of reported cases from May 21 to June 19 and the estimated generation time were used to estimate the effective reproduction number (Rt) by exponential growth method as summarized by Wallinga&Lipsitch [5].

## Results

### The basic situation of COVID-19 Delta Variant B.1.617.2 local outbreak in Guangdong

**Fig. 1:**
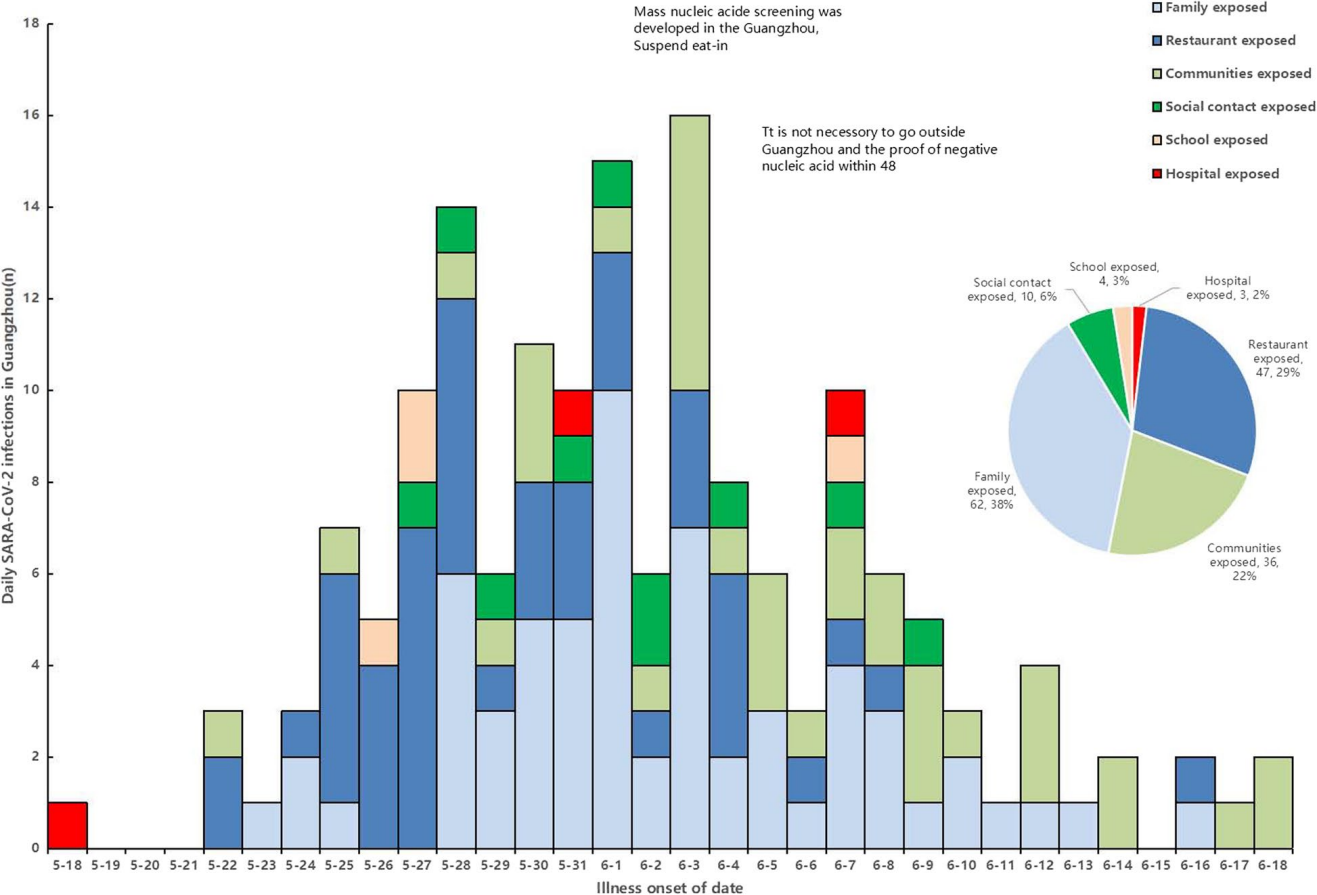
Epidemic curve of daily cases with graded and classified control interventions of the COVID-19 Delta Variant B.1.617.2 local outbreak in Guangzhou city, Guangdong Province, Southern China

21 May 2021, Guangzhou reported its first confirmed local case of COVID-19 Delta Variant B.1.617.2 infection, a 75-year-old woman who accidental exposure to an imported COVID-19 Delta Variant B.1.617.2 case in hospital. Subsequently, other local cases of this outbreak were identified, followed by chains of restaurant exposed, family exposed, communities exposed,school exposed, hospital exposed, social exposed. In order to contain the outbreak quickly, Guangzhou carried out graded and classified control measures in the places where cases occurred, including mass nucleic acid screening, travel restrictions, extensive contact tracing, epidemic control with health code, graded and adjusted management of communities according to risk levels, and the outbreak was contained quickly within two weeks (Fig. 1).

**Fig. 2:**
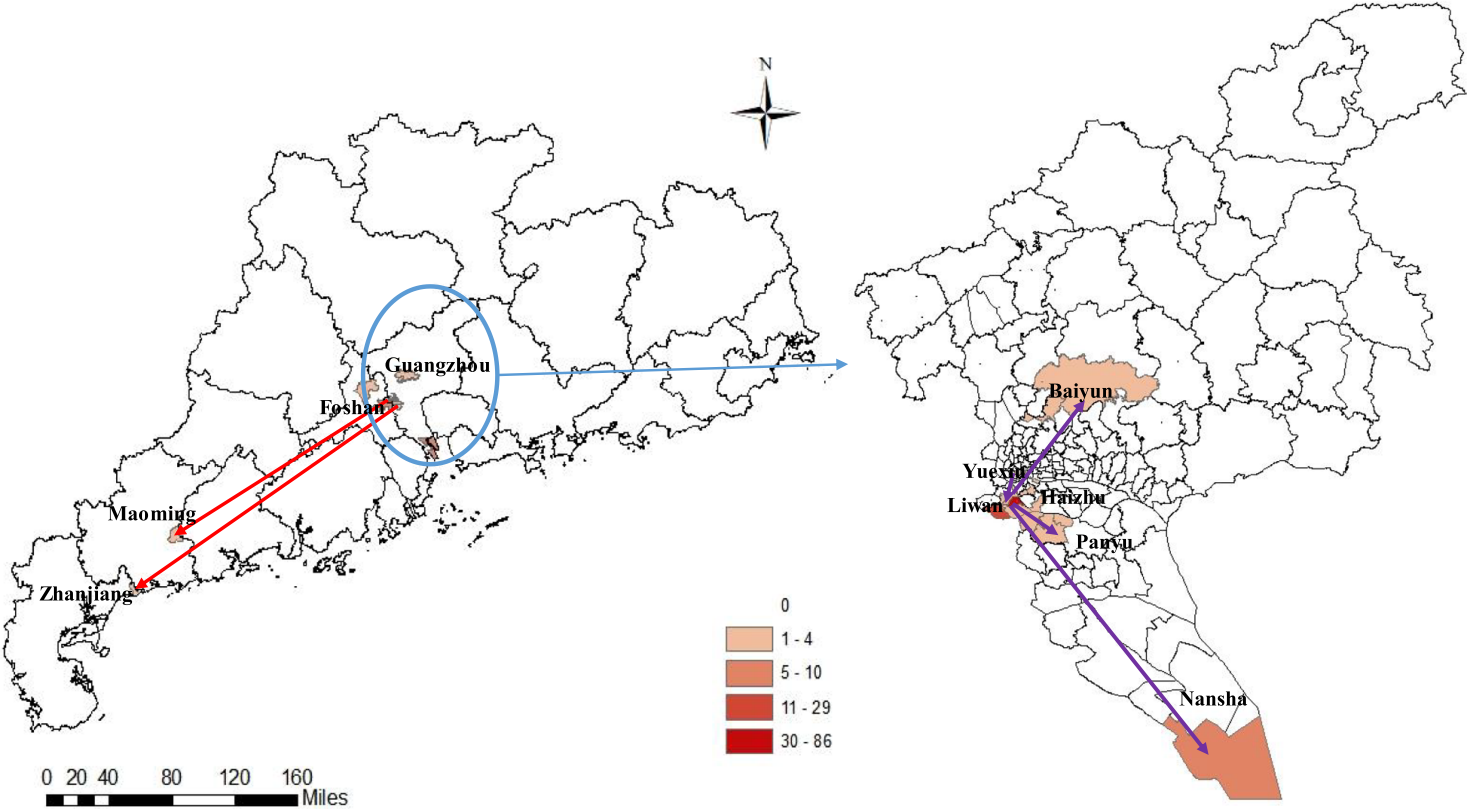
Distribution and flow direction of the COVID-19 Delta Variant B.1.617.2local outbreak in Guangzhou city, Guangdong Province, Southern China

A total of 161 cases were confirmed in the outbreak, with 147 cases detected in Guangzhou city and 14 cases spread to 3 other cities (Foshan city: 12 cases, Maoming city: 1 cases, Zhanjiang city: 1 cases) in Guangdong province.147 cases in Guangzhou city distributed in six districts of Guangzhou city, including Liwan district (122 cases), Nansha district (10 cases), Haizhu district (8 cases), Panyu district (4 cases), Baiyun district (2 cases), and Yuexiu district (1 cases) (Fig. 2).

### Incubation period, serial interval, generation time and the Rt in the COVID-19 Delta Variant B.1.617.2local outbreak

**Fig. 3:**
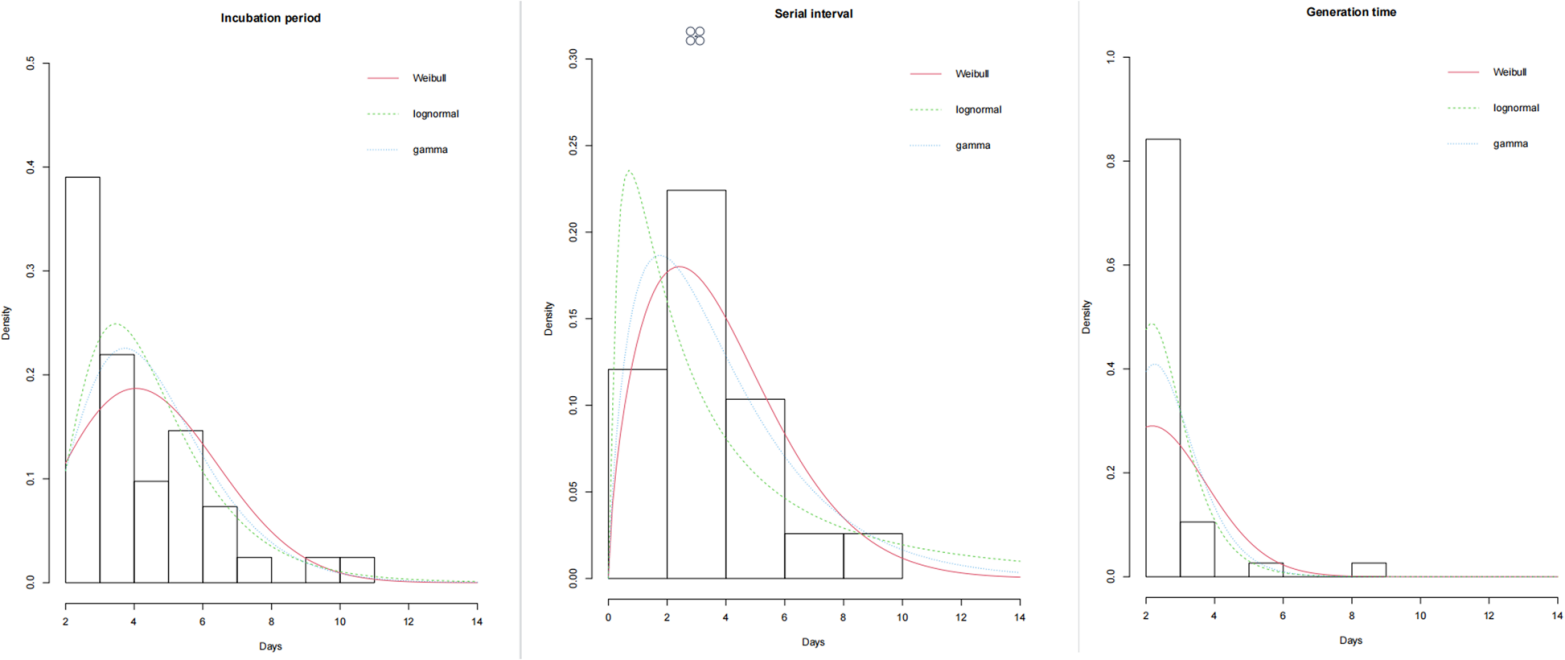
Incubation period, serial interval and generation time in the COVID-19 Delta Variant B.1.617.2 local outbreak

The optimal probability distribution of the corresponding incubation period, serial interval and generation time determined through AIC criterion were Lognormal, Weibull, Lognormal distributions, repectively. The estimated mean incubation period in the COVID-19 Delta Variant B.1.617.2 local outbreak was 4.22 (95%CI: 3.66–4.94) days, and the estimated 99th percentile ofincubation period was 9.28 (95%CI: 7.44–12.67) days. The estimated mean serial interval was 3.40 (95%CI: 2.61–4.30) days, and the estimated mean generation time was 2.60 (95%CI: 1.96–3.11) days. The estimated Rt for COVID-19 Delta Variant B.1.617.2 was 3.29 (95%CI: 2.25–5.07) (Table 1, Fig. 3).

**Table 1:** Incubation period, serial interval and generation time in the Delta Variant local outbreak

	**Mean(SD)**	**P**_**50**_**(P**_**25**_**-P**_**75**_**)**	**Estimated mean(95%CI)**	**99**^**th**^** percentile (95%CI)**
**Incubation period, days**	4.54(2.07)	4(3–6)	4.22(3.66–4.94)	9.28(7.44–12.67)
**Serial interval, days**	3.90(2.21)	3(3–5)	3.40(2.61–4.30)	—
**Generation time, days**	2.65(1.38)	2(2–3)	2.60(1.96–3.11)	—

### The total transmission chain of the COVID-19 Delta Variant B.1.617.2 local outbreak

From the first cases C1 who was detected in the fever clinic of a hospital in May 21^th^, to the last three cases C107,C156 and C157 who were transferred to be COVID-19 positive in the close contacts in June19th, a total chain of seven generations cases transmission occurred in the COVID-19 Delta Variant B.1.617.2 local outbreak. There were 1 case in Generation 1, 3 cases in Generation 2, 15 cases in Generation 3, 37 cases in Generation 4, 45 cases in Generation 5, 15 cases in Generation 6, 2 cases in Generation 7.

**Fig. 4:**
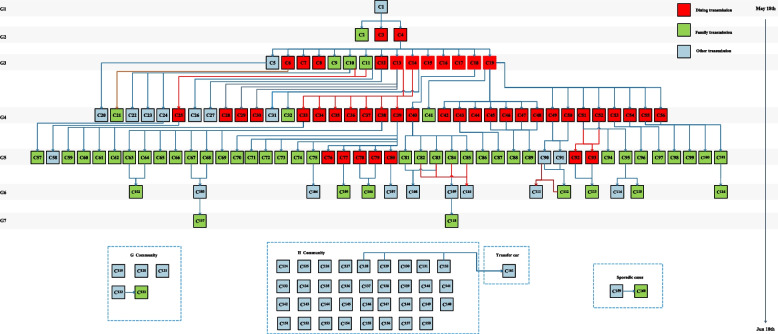
The transmission chain of the total 161 cases in the COVID-19 Delta Variant B.1.617.2 local outbreak

Epidemiological traceability showed that 118 of the 161 cases could be traced back to the previous communicator, another 40 cases occurred in G community and H community where cases were concentrated, 1 case(C161) was transmitted through the transfer car with cases C128, C129 and C132, and 1 sporadic cases(C159) were exposed in key areas where the cases had appeared, and then transmitted to her son C160.The transmission routes included dining exposure, family exposure, school and training exposure and other social activities exposure, among which dining and family exposure were the main transmission routes, with 47 cases (29.19%, 47/161) exposed through dining and 52 cases (32.30%, 52/161) exposed through family places (Fig. 4).

### Restaurant transmission chain in the COVID-19 Delta Variant B.1.617.2t local outbreak

**Fig. 5:**
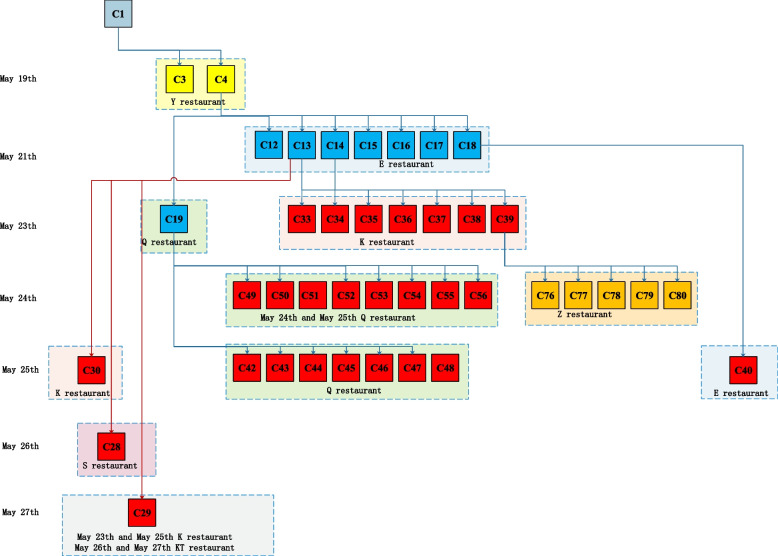
Restaurant transmission chain in the COVID-19 Delta Variant B.1.617.2t local outbreak

Residents in Guangzhou city were advised to take appropriate preventive measures during the period of Covid-19 prevention and control on an ongoing basis, including minimizing outdoor activities, avoiding crowded places, not organizing or attending parties or dinners, properly wearing masks when taking public transportation, entering supermarkets, shopping malls, hospitals, restaurants and other places, and keeping a safe distance of more than 1 m from others [6]. So people worn masks in most public places, except when dining and chatting at restaurants. Therefore,most of the early cases of theCOVID-19 Delta Variant B.1.617.2 local outbreak were transmitted through exposure in restaurants.C3, the waitress of Y restaurant was transmitted by C1 when served her on May 19th, and C4 was transmitted by dining exposure at the same time with C1.C4 spread to cases C12, C13, C14, C15, C16, C17, C18 by exposure together at E restaurant on May 21st, andspread to case C19 by exposure at Q restaurant on May 23th. Other restaurant transmitted cases were showed in Fig. 5.

### The community transmission chain in the COVID-19 Delta Variant B.1.617.2 local outbreak

There were 43 cases who could not be traced back to the previous communicator in the transmission chain, however most of them (93.02%, 40/43) lived in G community and H community where cases were concentrated.

Seventy-four cases (45.96%, 74/161) of the outbreak lived in the two communities, with 63 cases (39.13%, 63/161) in H community, and 11 cases (6.83%, 11/161) in G community.

Early cases in H community were infected from dining exposed on May 19th in Y restaurant, May 21st in E restaurant and May 25th in Q restaurant. With the subsequent family exposed, 28 cases in H community could traced back to the previous transmitter in the transmission chain. Cases C124 to C158 were the cases with no clear transmission chain in H community.

**Fig. 6:**
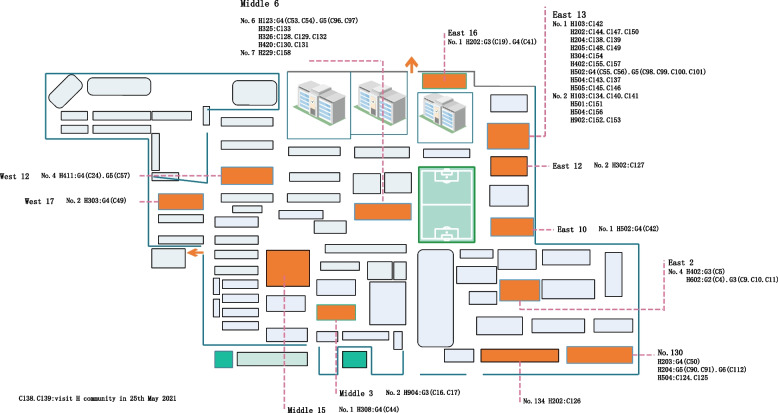
The distribution of cases in H community

May 26th, the H community implemented closed management and home quarantine of residents, but allowed family representative throw rubbish and got life supplies downstairs. And from June 3th, the management upgraded and required the residents to stay indoors at all time. Jun 7th to 8th, due to the continuous occurrence of cases, the residents who still quarantined in H community were transferred in batches to the isolation hotel. After that, the cases in H community were effectively controlled (Fig. 6).

**Fig. 7:**
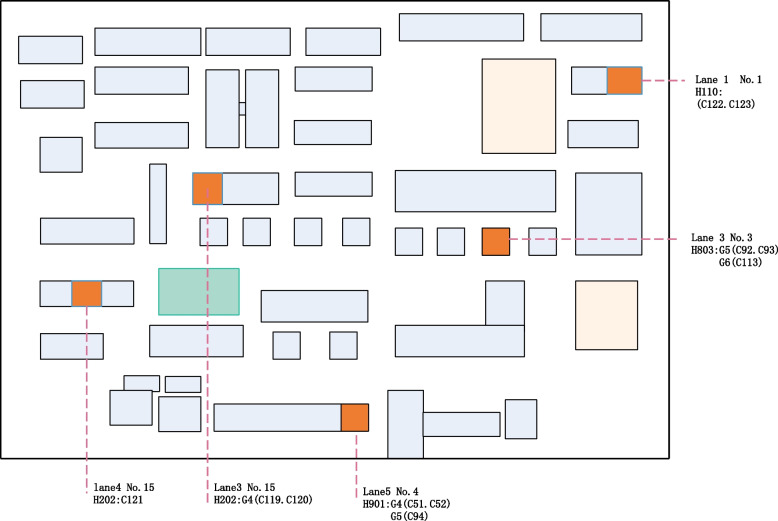
The cases distribution in G community

The first cases found in the G community were the family of C51, C52 and C94. C51 and C52 were a couple, and were infected by dining exposed in the Q restaurant on May 25th at the same time of case C19, and the infection was transmitted to their grandson C94 who lived with them. May 26th to 29th, C51 and C52 had breakfast in the breakfast shop which operated by C92 and C93, and transmitted the virus the C92 and C93. And the virus was next transmitted to C113, the family member of C92 and C93. In addition, G community also includes 5 sporadic cases (C119-C123) (Fig. 7).

### Superspreadersin the COVID-19 Delta Variant B.1.617.2 local outbreak

According to the SSEs definition, the 99th percentile of the offspring distribution for the number of secondary cases caused by a primary case in this COVID-19 Delta Variant B.1.617.2 local outbreak was 4.52, so we defined the SSEs in our study as individuals who were highly contagious and capable of transmitting a communicable disease to five and more than five cases of uninfected individuals.

**Fig. 8:**
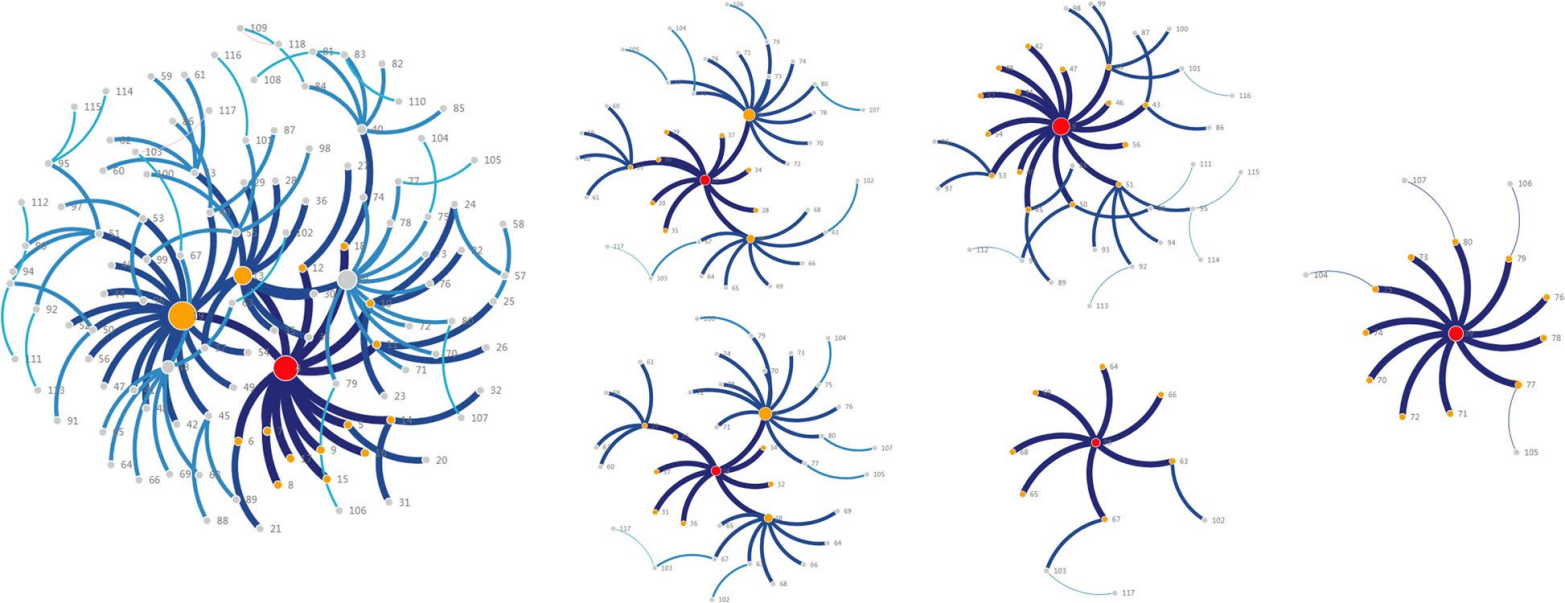
Superspreaders transmission chain

From the transmission chain, we could find 7SSEs who had more than 5 next generation cases (C4, C13, C14,C19, C38,C39and C40), and the 7cases and their infected cases infected 70.81%(114/161) of all the cases (Fig. 8). Dining exposure and family exposure were also the main transmission mode for the 7superspreaders.

Case C4, a 74-year-old female, retired people, infected 15 s-generation cases by dining and family exposure. C4 was infected by C1 on May 19th by the same time dining exposure in Y restaurant, and developed the disease on May 22th. And the infection was transmitted to C9 to C11 by family exposure, transmitted to C5 by daily contact in the same living building, transmitted to C6 to C8 by dining exposure in C6's house on May 22th, transmitted to C12 to C18 by the same time dining exposure in E restaurant on May 21st, and transmitted to C19 by the same time dining exposure in Q restaurant on May 23st.

Case C13, a 71-year-old male, retired people, onset date was May 25th, infected 10 s-generation cases by dining and daily social contact exposure. The infection was transmitted to C33 to C39 by the same time dining exposure in K restaurant on May 23th, transmitted to C30 by the same time dining exposure in K restaurant on May 25th, transmitted to C29 by dining exposure on May 23th and May 25th in K restaurant and on May 26th and May 27th in KT restaurant, and transmitted to C28 by the same time dining exposure in S restaurant on May 26th.

Case C14, a 58-year-old female, retired people, onset date was May 25th, infected 9 s-generation cases by dining and daily social contact exposure. The infection was transmitted to C33 to C39 by the same time dining exposure in K restaurant on May 23th, transmitted to C32 by visiting neighbors on May 23th, transmitted to C31 the doctors who treated her by nosocomial exposure.

Case C19, 85 years old, female, retired person, the onset date was May 25th, infected 16 s-generation cases by dining and family exposure. The infection was transmitted to C42 to C56 by the same time dining exposure in Q restaurant on May 25th,and transmitted to her grandson C41 by family exposure.

Case C38, 53 years old, female, retired person, the onset date was May 25th, infected 7 s-generation cases by family living and dining exposure. The infection was transmitted to C63 to C89 by family living exposure and multiple dinner on her daughter’s wedding ceremony.

Case C39, 56 years old, female, retired person, the onset date was May 28th, infected 11 s-generation cases by dining, family and daily social contact exposure. The infection was transmitted to C71 to C73 by family living exposure, transmitted to C74 and C75 by visiting neighbors on May 26th, transmitted to C70 by dancing together on May 256th, transmitted to C76 to C80 by the same time dining exposure in Z restaurant on May 25th.

Case C40, 34 years old, female, housewife, the onset date was June 1st, infected 5 s-generation cases by family living. The 5 s-generation cases were all family members, including her husband C81, father-in-law C82,mother-in-law C83, two daughters C84 and C85.

## Discussion

As the COVID-19 pandemic goes on, there has been a mutation evolution of SARS-CoV-2 [7]. And the pathogen leaded the Guangzhou COVID-19 local outbreak in 2021 was the COVID-19 Delta Variant B.1.617.2.

Researches show that, the mean incubation day and the mean serial interval day in Wuhan COVID-19 outbreak in Dec 2019 were 5.2 days and 7.5 days of early cases [8], while the median of incubation period and the median of serial interval between primary case and secondary cases in the Beijing clusters of COVID-19 in Jun 2020 were all 5 days [9, 10]. The Delta B.1.617 was more virulent, the virus transmission ability was stronger, and the incubation period is shorter. Our study shows that, in the Guangzhou 2021 COVID-19 Delta Variant B.1.617.2 local outbreak, the mean incubation period was 4.22 days, and the serial interval was 3.40 days, the mean generation time was 2.60 days.

Through our research, we found three factors mainly made this outbreak spread, dining and family exposure, super spreaders, and the community transmission.

There are 29.19% cases infected by dining exposure and 32.30% cases infected in family places, so dining and family exposure were the main transmission route of the outbreak. With the habit of having breakfast in the morning tea restaurants of the local residentsespecially the senior citizenin Guangzhou city, most early cases of the outbreak were retired people who have the morning tea habit and along with a number of family members living together, they caused the rapid spread of the outbreakand brought greater difficulty for epidemic control and prevention.

Short-range airborne transmission was currently recognized as a predominant route for transmission of SARS-CoV-2, however, the CDC had also acknowledged the importance of transmission of COVID-19 through inhalation of viruses in the air at distances farther than six feet (2 m). Several important factors contributed to increase risk, including enclosed space with inadequate ventilation, increased exhalation of respiratory fluid such as shouting, singing, and exercise, and prolonged exposure more than 15 min [11]. Crowded indoor environments such as households, were high-risk settings for the transmission of SARS-CoV-2. Katlama C, etc. evaluated the prevalence of SARS-CoV-2 antibodies in 87 locked-down families households to determine viral dynamics and immunity acquisition, and concluded that households represent a high-risk setting for SARS-CoV-2 transmission through close contact within the family amplified by the number of family members and the housing surface area [12]. And dining settings in public spaces were considered have more risk of SARS-CoV-2 transmission, due to the diners could not wear masks while dining and need to share the space with other diners. The CDC study found that implementing mask regulations was associated with reduced transmission of SARS-CoV-2, implementing mask mandates was associated with reduced SARS-CoV-2 transmission, whereas reopening restaurants for on-premises dining was associated with increased transmission [13]. Li Y, etc. analyzed assessed the possibility of airborne transmission in a COVID-19 outbreak involving three families in a restaurant in Guangzhou, China, the results showed that the measured averaged ventilation rate of 0.90 L/s per patron in the restaurant is considerably lower than the 8–10 L/s per person required by most authorities or professional societies, and suggested that crowded and poorly ventilated spaces are more likely to lead to the spread of SARS-CoV-2 [14].

Super spreaders compared with general infection are more likely to infect others. Reference show that 19%of cases seeded 80% of all local cases between 23 January and 28April 2020 in Hong Kong [15], and our study show that4.35%of cases seeded 70.81% of all cases in this outbreak.

In our study, 45.96% cases of the outbreak lived in the same communities. H community and G community located on the opposite side of the same road, and shared living facilities such as restaurant and supermarkets. Most residents of the two communities were retired workerswho worked in the factories years ago and formed the habits of dining together and visited each other so often., the above factors lead to the further spread of the outbreak in the two communities.

The incubation period is essential for infectious source tracing, the appropriate duration of quarantine of close contacts, and carried out control measures. Our results showed that the estimated mean incubation period was 4.22 days in the Guangzhou 2021 COVID-19 Delta Variant B.1.617.2 local outbreak, compared to the systematic review and meta-analysis results from the data source that searched between December 1 2019 and February 10 2022, the mean incubation period of Delta variant B.1.617.2 in our research was consistent with the results of Delta variant in the meta-analysis (4.41 days, 95% CI 3.76–5.05 days), and shorter than the value of Alpha variant (5.00 days, 95% CI 4.94–5.06 days) and Beta variant (4.50 days, 95% CI 1.83–7.17 days), longer than the value of Omicron variant (3.42 days, 95% CI 2.88–3.96 days) [16]. The estimated 99th percentile ofincubation period(9.28 days) in the Delta outbreak suggests the infectious sourcetracing and quarantine period of 2 week are deemed more suitable, which is consistent with the practice of quarantine period in the Alpha outbreak [17]. In the outbreak, the mean estimated serial interval (3.40 days) was shorter than pooled mean serial interval of 3.9 daysfor Delta and longer than the value of 3.2 days for Omicron (20 studies) [17, 18]. The mean estimatedgeneration time (2.60 days)was shorted than the value of 5.20 daysin Singapore and 3.95 days in Tianjin for Alpha [19]. However, the estimates for othervariants have been rarely published since the generation time is seldom observable [20]. We also found that the mean estimated serial interval (3.40 days)and generation time(2.60 days)wereshorterthanthe mean incubation period (4.22 days)which indicated the possibility of presymptomatictransmission of COVID-19 Delta Variant B.1.617.2.

The early stage of the outbreak was linked to the initial reproduction ratio. The estimated Rt (3.29) of our research wasmuchhigherthan the value of 2.2 in Wuhan city [8], which suggested that COVID-19 Delta Variant B.1.617.2 has a potentially higher rate of transmission than other variants. Evidence is accumulating to indicate a highly contagious infection of COVID-19 Delta Variant B.1.617.2 [21, 22].

## Conclusions

As the first Delta Variant local outbreak in China, Guangzhou upgraded the prevention and control mode. Classification and dynamically adjusted prevention and control measures had been carried outaccording toanalysis of transmission chains and epidemicalrisk levels, including promoting nucleic acid screening at different regions and different risk levels, dividing closed-off area, controlled area according to the risk of infection, raising the requirements of leaving Guangzhou. By the above control measures, Guangzhou effectively control the outbreak within 28 days without implementing a large-scale lockdown policy.

## Data Availability

The data that support the findings of this study originated from Guangzhou Center for Disease Control and Prevention. And most data in the study were not publicly available due to derived from epidemiological investigation reports that are not publicly available, so data will be available on reasonable request by email to the corresponding author.
